# Guided self-study in preclinical physiotherapy students – A feasibility study

**DOI:** 10.4102/sajp.v79i1.1866

**Published:** 2023-10-12

**Authors:** Elisabeth Schenk, Jan Taeymans, Slavko Rogan

**Affiliations:** 1 School of Health Professions, Division of Physiotherapy, Bern University of Applied Sciences, Bern, Switzerland; 2 Faculty of Physical Education and Physiotherapy, Vrije Universiteit Brussel, Brussel, Belgium

**Keywords:** higher education, learning gain, self-study, teacher-centred instruction, self-directed learning

## Abstract

**Background:**

Literature describing the impact of guided self-study (G-SS) in knowledge changes and skills improvements in undergraduate students is scarce.

**Objectives:**

The aims of our study were to evaluate the feasibility of a G-SS programme in a full-time undergraduate physiotherapy degree course and to assess the effectiveness of the G-SS on changes in knowledge and development of skills (hands-on).

**Method:**

Fifty-three first-semester undergraduate physiotherapy students were randomly divided into a G-SS group and a control group (CG). The G-SS group received six clinical cases and prepared each case during an 8-day cycle. The control group received self-study learning units of the original curriculum content. Primary outcome parameters were (1) time of task, (2) responsiveness of students and (3) programme differentiation. Knowledge changes and skills changes were tested using a multiple-choice questionnaire and the objective structured clinical examination (OSCE).

**Results:**

Students’ responsiveness was 32%. This was below the a priori set 83%. No differences in programme differentiation were found. The OSCE grade was significantly higher in the G-SS compared to CG (*p* = 0.003).

**Conclusion:**

The G-SS programme in its current form was not feasible regarding students’ responsiveness. Therefore, a slight modification of our study protocol (e.g., better time planning in the academic calendar) is needed to improve students’ willingness to participate in the G-SS programme.

**Clinical implications:**

Adaptation of the school timetable should allow undergraduate physiotherapy students to prepare clinical cases under conditions of lower workload. Guided self-study as compared to CG is superior in knowledge change and (hands-on) skills improvement.

## Introduction

Self-study is an important component in higher education settings (Herren 2014; Landwehr & Müller 2008). The literature postulates two forms of self-study: free self-study (F-SS) and guided self-study (G-SS) (Landwehr & Müller 2008; Rogan 2015). During F-SS, students are self-reliant. During G-SS, students are supported by lecturers (Rogan 2015). While F-SS has been incorporated in the physiotherapy curricula at the Bern University of Applied Sciences (BFH) for many years, G-SS should be planned and implemented based on scientific evidence.

Landwehr and Mueller (2008) and Rogan (2015) postulated a standardised procedure to implement G-SS in higher education curricula.

Landwehr and Mueller reported five phases for G-SS (Landwehr & Müller 2008). In Phase 1, the preparatory stage, students receive an assignment (clinical case description) from the lecturer with clear-cut learning objectives. In Phase 2, students work in small groups on their learning assignment with coaching and support from the lecturer. Phase 3 consists of a guided plenary session in which students present an insight into their learning outcomes to the tutor and their peers. Students and lecturer reflect on the learning process during Phase 4. In Phase 5, students receive feedback on their presentations and learning processes from their peers and lecturer.

Based on this didactic model, Rogan et al. (2020, 2021) investigated the feasibility and effects of G-SS on the learning gain of practical physiotherapy skills in undergraduate physiotherapy students at the BFH. The first randomised controlled feasibility study (Rogan et al. 2021) demonstrated that an adaptation of the timing of G-SS in the school schedule is needed to increase students’ responsiveness to the G-SS units in the first semester. Several students (self-study group, *N* = 26) who had experienced G-SS passed all exams, while 4 out of 25 students from control group (CG) (i.e., F-SS) failed. Furthermore, students from the G-SS group demonstrated significantly better exam grades compared to students from the CG. In the second randomised controlled study (Rogan et al. 2020), the feasibility of implementing three G-SS units and the effectiveness on the learning gain of BSc physiotherapy students were assessed. It was recommended that the study design should be adapted regarding the scheduling of G-SS during periods with a low workload for the students. There was, however, no difference between the groups in terms of learning gain.

This feasibility study is part of the larger, longitudinal ‘Retired PhysioTherapists’ Tutor Supported Learning’ study (RePTusule), which is conducted at BFH. The RePTusule study investigates how retired physiotherapists can act as tutors in G-SS, how this tutoring can affect the age image and the learning gain of physiotherapy students, and if this tutoring has a protective effect against age-related reduction of physical and cognitive capacity in the participating retired physiotherapists. The involvement of retired physiotherapists as tutors in the G-SS, which was added in this pilot study in comparison to the previous studies by Rogan et al. (2020, 2021), has the objective to be able to better guide all small groups individually as well as to bring in the longstanding professional experience and expertise of the retired physiotherapists.

The primary aim of our study was to assess the feasibility of fidelity of implementation. In addition, we wanted to demonstrate the impact of six G-SS units on the learning gain of physiotherapy students in their first semester, in comparison to the F-SS that has taken place to date at the BFH. The research questions of our feasibility study were as follows:

- Is it possible to conduct a guided self-study with retired physiotherapists among physiotherapy students at the BFH in the first semester?
- Is there a difference in learning gain among students of cohort PHY19 between participation in the G-SS and free self-study (CG) at the end of the first semester in the module ‘Basics of Clinical Physiotherapy Examination’?

## Method

### Study design, setting, quality reporting and ethics

**FIGURE 1:**
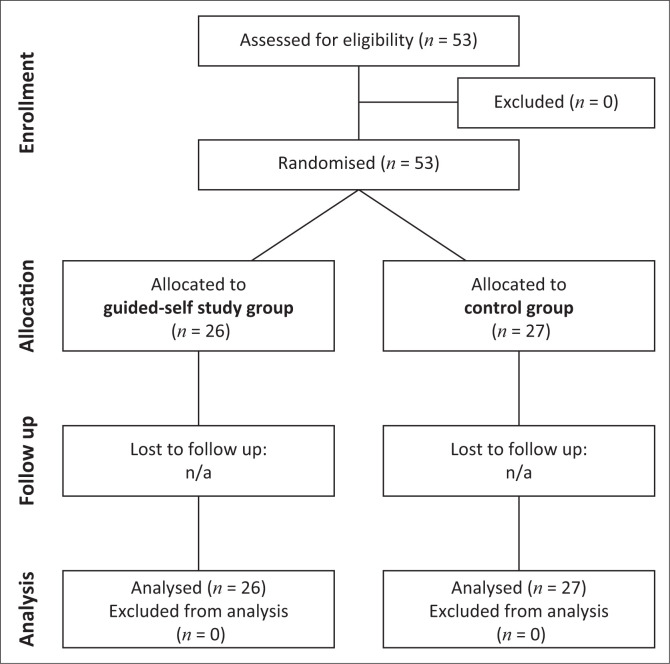
Study design.

Our cohort randomised feasibility education study was designed as a prospective, single-centre, two-arm study and was conducted at BFH Department of Health, Division of Physiotherapy. Our article was written following the CONSORT 2010 checklist (Hopewell et al. 2008). Figure 1 presents the flow of our feasibility study.

### Participants and recruitment

Young healthy physiotherapy students (between 18 and 25 years of age) of the undergraduate physiotherapy degree course PHY19 from the first semester (entry level) of the BFH and retired^1^ physiotherapists were invited to volunteer in our study. There are numerous classes for bachelor’s degree courses in physiotherapy. Candidates must perform a two-stage professional individual suitability test. Candidates must have an *Abitur*, or vocational school-leaver’s certificate, and the best 104 candidates are selected for the full-time physiotherapy degree course. All students fulfil the same requirements in terms of school-leaving qualifications and previous knowledge. Because of this procedure, the group was still homogeneous in terms of knowledge and skills at the beginning of our study. Exclusion criteria were undergraduate physiotherapy students of the physiotherapy study bachelor’s degree course 2018 who needed to repeat the first semester and students from other BFH degree courses or from other institutions. Furthermore, retired physiotherapists who were still clinically active at a level of more than 10% were also excluded.

Recruitment of undergraduate physiotherapy students of the course PHY19 (2019) for our feasibility study was carried out by means of an oral information session with the distribution of declarations of consent. The potential study participants were given 2 weeks to decide on volunteering. Retired physiotherapists were recruited through an announcement in the official journal of the Swiss Physiotherapy Association, *Physioactive*, and by asking colleagues. All participants provided written informed consent.

### Randomisation

Randomisation of physiotherapy students into groups A, B, C and D is a standard procedure at BFH to keep the group size for practical lessons small (< 15) and to foster group learning. Groups A and B and groups C and D are together in seminars and workshops. This allocation of groups A and B and groups C and D is then fixed for the first semester. For the next semester, the groups are reassigned.

An independent researcher conducted a computer-generated randomisation that assigned the students of groups A and B and the students of groups C and D to a tutor guided self-study groups (G-SS-G; *n* = 27) and a group of students participating in free self-study as the control group (CG; *n* = 28) as planned in the traditional curriculum of the bachelor’s degree course. The students from the G-SS-G were then divided into four groups of 6–7 students each, which were supervised or guided by a tutor during the 8-day cycle.

### Intervention

The G-SS concept was based on the previous investigations by Rogan et al. (2015, 2020, 2021): a total of six G-SS periods were scheduled between the start of October 2019 and the start of January 2020. The G-SS period consisted of an 8-day cycle. A clinical case was processed in each G-SS session. In total, 6 weeks were scheduled. Clinical cases were used for the G-SS sessions that aligned with the module contents of the undergraduate physiotherapy degree programme. The clinical cases were not targeted to the semester exam. Table 1 presents an overview of the contents of the clinical cases in the tutor G-SS sessions. Each of the four groups had a tutor who guided the group during the 8-day cycle.

**TABLE 1:** Overview of the guided self-study clinical cases proposed in 8-day cycle procedure.

G-SS period	Clinical case	Learning objective
1	Thoracic massage of an elderly person after heart surgery.	To perform massage techniques on two different positions. To develop a massage checklist.
2	Colleague with a muscle stiffness in the region of the hamstring after squash.	To develop an examination protocol. To explain a physiological reflex model of muscle stiffness.
3	Gait analysis of an elderly person and younger person.	To develop a gait analysis checklist. To develop an examination protocol for gait analysis.
4	Measurement of body joint angles with goniometer and cellphone-based applications.	To explain the differences between the neutral-zero measurement method and cellphone-based applications. To develop a checklist for traditional joint angle measurement for hip and knee joint mobility.
5	Passive and active joint examination, translational joint examination and tests for muscle flexibility and muscle strength of the pelvis-hip region.	To perform a specific examination of the hip region in a timeframe of 8 minutes.
6	Football player with knee pain, with a pain area around the adductor tubercle.	Hypothesis-deductive approach to an examination of the lower extremity.

G-SS, guided self-study.

**FIGURE 2:**
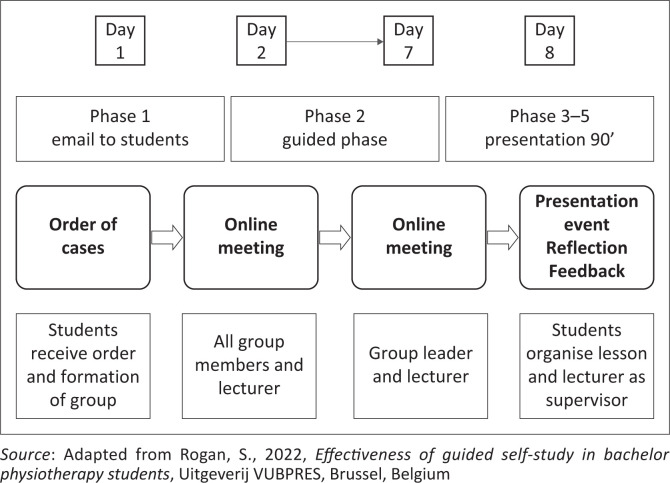
Process flow chart of the 8-day cycle.

On day 1, each of the four groups was informed about the learning goals and received the clinical case description and corresponding tasks as well as contact details of the tutor via email (Phase 1). From day 2 to 7, every individual group could choose between a tutor-supported approach (e.g., online meeting or meeting onsite in a lecture hall) or not (Phase 2). On day 8, every individual group presented the results of their work to the tutor and to their peers in the lecture hall (Phase 3). Students carried out an oral reflection on their work (Phase 4). At the end of day 8, each tutor moderated a plenary session in the group including feedback (Phase 5). The duration time of the session on day 8 was 90 min. The higher education lecturer from BFH moderated this 90-min session (Phases 3–5). Figure 2 depicts the flow of the 8-day cycle intervention.

### Control group

Students of the CG were self-reliant in F-SS and performed F-SS as planned in the traditional curriculum of the bachelor’s degree course.

### Outcomes

#### Primary outcome: Feasibility

Feasibility was assessed as ‘fidelity of implementation’ (Lastica & O’Donnell 2007; Mowbray et al. 2003), which was evaluated as follows:

- *Time of task* included the total number of the conducted G-SS sessions and the duration of each G-SS session in minutes.
- *Students’ responsiveness*: Tutors documented students’ presence in the attendance list after each G-SS session (Phases 3–5) and by a post-intervention oral group interview survey. An adequate responsiveness to the study protocol was defined as every physiotherapy student of the G-SS group having attended five out of six G-SS sessions, with 83% of students consenting (Rogan et al. 2021).
- *Programme differentiation* was evaluated during the programme conception. Programme-related and programme-competing content in the G-SS cases and the curriculum were investigated.

#### Secondary outcome: Impact of intervention

Students’ learning outcomes were evaluated using Kirkpatrick’s model (Chrysafiadi & Virvou 2013; Diefes-Dux et al. 2004; Kirkpatrick 1998). Kirkpatrick’s model consists of four levels: Level 1: reaction; Level 2: learning; Level 3: behavioural changes; Level 4: organisational performance. It is usually not possible to measure all four levels at once (Embi, Neo & Neo 2017). Therefore, only level 2 was implemented in our feasibility study. Physiotherapy students of the bachelor’s physiotherapy programme at BFH were evaluated twice per semester for written (multiple-choice questionnaire [MCQ]) and practical (objective structured clinical examination [OSCE]) competences. Multiple-choice questionnaire was scored on a maximum of 87 points and converted into a grade. The OSCE consisted of eight stations with a total score of 48 points (i.e., 6 points per OSCE station). Students passed both exams if a score of 60% was reached.

#### Statistical analysis

Data were analysed with descriptive statistics and presented as means with corresponding standard deviations (s.d.) as well as by medians with interquartile ranges (IQR).

An intention-to-treat (ITT) analysis was performed, to guarantee that the randomisation remains unbroken. Missing data were replaced by the median values of the group to which participants were originally allocated (Hollis & Campbell 1999). To determine differences in the exam results between the G-SS-G and CG after the first semester, the Mann-Whitney U test was used using the exact sampling distribution of U (Dinneen & Blakesley 1973). All calculations were performed using the Statistical Package for Social Sciences (SPSS) version 27.0 (IBM Corp. Released 2020. IBM SPSS Statistics for Mac, Version 27.0. Armonk, New York, United States: IBM Corp.)

### Ethical considerations

Ethical approval was granted before starting this feasibility study, and the ethics committee of the Canton of Bern, Switzerland (No. 2018-01683) gave its approval. Our study was also registered at the Deutschen Register Klinischer Studien (DKRS: DRKS00015518).

## Results

A cohort with a total of 53 students and two retired physiotherapists (tutors) was included in this feasibility study. As originally intended, in order to cover all groups with one tutor, additionally two physiotherapists were recruited. One had 20 years of working experience, and held an Orthopaedic Manual Physical Therapy (OMPT) degree and an MSc Physiotherapy degree. The other tutor was a higher education lecturer from the university with 19 years of teaching experience. Table 2 gives an overview of students’ characteristics.

**TABLE 2:** Overview of students’ characteristics.

Variables	G-SS	CG	Total
*N*	26	27	53
Age (years) (mean ± s.d.)	22.19 ± 3.37	21.30 ± 2.13	21.74 ± 2.82
Men (*n*)	6	5	11
Women (*n*)	20	22	42

G-SS, guided self-study; CG, control group.

### Primary outcome

#### Feasibility

**Fidelity of implementation of time of task:** all six units of the G-SS were conducted as planned. The a priori set time limit of maximum 90 min for the plenary session at day 8 was not exceeded.

**Fidelity of implementation of students’ responsiveness:** the 83% target of five attendances per student was not achieved (Table 3).

**TABLE 3:** Overview of guided self-study students’ attendance on presentation day (day 8) of each cycle.

Case	Number of students during day 8			
	In attendance	Missing	Total	In attendance (%)
Case 1	24	2	26	92.31
Case 2	23	3	26	88.46
Case 3	16	10	26	61.54
Case 4	13	13	26	50.00
Case 5	12	14	26	46.15
Case 6	12	14	26	46.15

**Total**	**100**	**56**	**156**	**64.10**

Post-intervention interviews showed that the feedback from the tutors on the G-SS was positive. The topics were well aligned with the module content and the learning outcomes. The questions in the cases were not too difficult, but some students struggled to see the clear aim of the task from the beginning. Unfortunately, the G-SS was not well integrated into the schedule. The total workload of the students in the semester was too high. The supervision by the tutors was found helpful, practical and interesting by the students. The broad perspective of the tutors and the overview they brought with them was felt to be overwhelming for some students: ‘how are we supposed to know all this […]’, and ‘we are only in the first semester’.

The time required in the 8-day cycle to complete the case was mentioned as suitable. The preparation time was considered appropriate, as was the 8-day cycle as such. However, because of the high overall workload in the study course, it was not always possible for the students to prepare the case completely.

All 26 students from the G-SS and 25 out of 27 students from the CG passed the written MCQ exam. All students from both groups passed the OSCE. An ITT analysis was performed for all OSCE and MCQ values. Table 4 shows significant group differences between the G-SS and CG for OSCE 5 times (*p* < 0.0001) and OSCE 6 times (*p* < 0.0001). Table 4 also shows an overview of MCQ and OSCE total scores in the median and interquartile range (IQR). The findings of ITT analysis are shown.

**TABLE 4:** Multiple choice questionnaire and objective structured clinical examination scores in median and interquartile range (IQR).

Form of examination	G-SS (*n* = 26)		CG (*n* = 27)		*p* (5 sessions)	*p* (6 sessions)
	Median	IQR	Median	IQR		
OSCE	4.48	4.35–4.61	4.58	4.28–4.65	0.0001*	0.0001*
MCQ	4.38	4.11–4.60	4.54	4.32–4.65	0.126	0.266

MCQ, multiple choice questionnaire; OSCE, objective structured clinical examination; G-SS, guided self-study; CG, control group; IQR, interquartile range.

* , *p* = < 0.05 (Mann-Whitney *U* Test).

## Discussion

Our higher education feasibility study aimed to assess the feasibility and effectiveness of retired physiotherapist tutors supporting self-study among first-semester physiotherapy students at a Swiss University of Applied Sciences.

The first research question focusing on feasibility was formulated as ‘is it possible to conduct eight cycles of guided self-study supported by retired physiotherapists as tutors among first semester undergraduate physiotherapy students of a Swiss University of Applied Sciences?’

Our findings showed that the preparation time for the case was considered appropriate, and the time needed to process the case in the 8-day cycle was described as suitable. However, because of the high overall workload in the study course, it was not always possible to prepare the case completely. Therefore, it is not possible to implement the planned, guided self-study at the undergraduate physiotherapy programme in its current form. A modification of our study design is needed to increase students’ willingness to participate in all G-SS sessions. The willingness to learn and subsequent engagement in learning reflect the choice of educational task. Empirical research shows that the subjective value of a task predicts the choice of task (Harackiewicz et al. 2008). Gorges, Schwinger and Kandler (2013) explained that the value of a task is influenced by the recollection of previous motivation to learn. In addition, the learner’s self-concept is another component that is also closely linked to task value. It is assumed that important factors influencing the formation of task value are the learner’s goals, task-specific self-concept and interpretation of past events (e.g. attribution of success and failure) (Gorges et al. 2013). These are in turn influenced by socialisation, education and upbringing. Learners themselves as well as the institution could benefit from an explicit consideration of recalled educational experience. The adoption of maladaptive performance avoidance goals on negative performance experiences (Anderman & Maehr 1994) could be reshaped through altered attributions as happens in trauma therapy (Van der Hart et al. 1993).

Therefore, curriculum planners should schedule the G-SS cycles in periods with lower workload for the students, for example, avoid G-SS in the week before an exam period. The criteria of success for the fidelity of implementation of time of task were a priori set at 83% participation in five out of six units of the G-SS and a planned duration time of maximum 90 min for the plenary session at day 8. Eight out of 26 students (32%) instead of the expected 83% participated at day 8 of at least five G-SS cycles. The first and second cycles were very well attended (> 88%), because these two cycles were scheduled in a time period with a workload for the students below 40 h per week. Between the first and the last G-SS cycle, participation decreased by 46%. According to the students participating in the post-intervention interviews, the workload was very high from cases three to six. Another reason was that during November 2019 and December 2019, a higher learning workload was scheduled by the programme planners to allow students to receive more learning time to prepare themselves in January 2020 for the exam in February 2020. In addition, students’ workload increased in December 2019 because of the completion of a thesis project. The scheduling of the G-SS in the school timetable was reported by the physiotherapy students as the main reason for the observed low participation. These findings were similar to the studies conducted by Rogan et al. (2020, 2021). Newble and Entwistle (1986) mentioned in their article that student learning is influenced by external circumstances and by individual characteristics. It is well known that a higher number of teaching hours leads to a higher workload for students, which prevents deeper learning and tends to lead to superficial approaches to learning (Kember 2004). Findings from higher education studies suggested that instruction time above 20 h per week leads to reduced self-study time (Credé, Roch & Kieszczynka 2010; Kember et al. 1996). Biggs (1979) and Entwistle (2013) showed that other individual characteristics such as the predominant motivation of a student to the achievement of high grades and students’ feeling of competition were also important factors in their decision-making process whether to choose deep learning or superficial learning. Learning workload for exams presents an example of such external learning circumstances. Exams can be strong stimuli for learning (Wieland 2016). These factors must be considered when scheduling G-SS in the timetable.

Our study was able to obtain the same feedback from physiotherapy students in relation to the content of the cases and goals of the cases that were aligned with the module content in the same manner as Rogan et al. (2020, 2021) postulated in their feasibility studies. This circumstance promotes the willingness of undergraduate physiotherapy students to accept clinical cases and not to reject them a priori. The cases (Table 1), therefore, can be used in the same format in our next study.

The second question focused on the effectivity of G-SS on learning and was formulated as follows: Is there a difference in learning gain among students of cohort PHY19 between participation in the G-SS and free self-study (CG) at the end of the first semester in the module Basics of Clinical Physiotherapy Examination? The answer was that guided self-study was appropriate to consolidate practical (hands-on) skills in undergraduate students in the first semester.

Peer-Assisted Learning (PAL), during which older students are enabled to facilitate younger students in teaching and learning, is being used in many undergraduate and postgraduate programmes (Topping & Ehly 1998, Whitman & Fife 1988). Higher education research in the fields of nursing, medicine and physiotherapy reported learning benefits in laboratory and clinical education (Aston & Molassiotis 2003; Aviram et al. 1998; Escovitz 1990; Hammond et al. 2010; Sevenhuysen et al. 2017). Ologunde and Rabiu (2014) described PAL as ‘People of similar social groupings who are not professional teachers helping each other to learn and learning themselves by teaching’. In our feasibility study, the tutors were two retired physiotherapists and two physiotherapists with clinical experiences. We found that all students in the G-SS group but only 25 out of 27 students from the CG, following the original curriculum content with individual self-study, passed the written MCQ exam.

In the meta-analysis by Murad et al. (2010), findings from 59 studies, totalling 8011 health-profession students, were compared on the effect of traditional learning methods versus self-directed learning (SDL) in the knowledge domain. Self-directed learning showed significant gain in factual knowledge, but not in skills. However, if students are involved in the selection of learning materials, SDL seems to be more successful. For this reason, teachers should support learners in self-study and involve them in the selection of suitable learning materials (texts, videos, etc.). University lecturers should involve undergraduate physiotherapy students by selecting suitable learning materials for future studies.

Moreover, Murad et al. (2010) concluded that SDL is more successful with experienced students. In our study, the participants are undergraduate physiotherapy students in their first semester. In future, it would be appropriate to introduce the students to SDL at the beginning of the first semester. Self-directed learning is described as the process of learners managing their learning on their own initiative, identifying their learning needs, formulating learning objectives, determining the essential material and personal resources for their learning, selecting appropriate learning strategies and evaluating learning outcomes.

### Study limitations

A limitation of our feasibility study was the interview with the students after the last G-SS unit at case six. In this survey, 12 out of 26 (46.15%) students participated. Therefore, only a few participants were able to give feedback. An anonymous quantifiable questionnaire that could be sent to all participants could increase the response rate. This would provide measurable and comparable data. The inclusion criteria for the retired physiotherapists were very strict and allowed only two retired physiotherapists to participate. As a result, an additional Master of Physiotherapy with 15 years of working experience and OMPT degree and a higher education lecturer were used to cover all groups. Prior to the start of the programme, a joint review session was held on the didactic concept of guided self-study and the role of tutoring. However, students expected retired physiotherapists, and the mix of tutors may have caused bias or even negatively influenced personal attitudes towards the programme. This may have had an impact on students’ attitudes, responsiveness or even engagement. The reason for the low recruitment rates was that a maximum occupation level of 10% has been provided for retired physiotherapists. Physiotherapists of this generation did not pay a lot into the retirement and survivors’ funds. Consequently, their pensions are small. Therefore, physiotherapists continue to work longer and with higher workloads to supplement their pension income. This was communicated to our study leader during recruitment by the retired physiotherapists.

## Conclusion

Our feasibility study demonstrated a G-SS programme that has been conducted for first-semester undergraduate physiotherapy students at the BFH in Switzerland. The findings show that the selected study design was not feasible in its current form. An adaptation of our study design must be conducted for future studies. All six G-SS sessions were carried out as scheduled. Students’ responsiveness to G-SS sessions was low at 32%, in comparison with the expected 83%. The G-SS sessions that were scheduled during the period of high workload were considered to be the main reason for the low student attendance. Upcoming higher education studies must take into account students’ workloads when planning the G-SS session.

Guided self-study provides favourable impacts in knowledge changes and skills improvements when undergraduate physiotherapy students prepare and present all clinical cases and give feedback as a reflection process at the end of an 8-day G-SS cycle.

## Footnotes

1 Also retired physiotherapists who still work in the practice for a maximum of 10%.
